# Efficacy of violet–blue light to inactive microbial growth

**DOI:** 10.1038/s41598-022-24563-1

**Published:** 2022-11-23

**Authors:** Davide Amodeo, Valentina Lucarelli, Isa De Palma, Alessandro Puccio, Nicola Nante, Gabriele Cevenini, Gabriele Messina

**Affiliations:** 1 Department of Medical Biotechnologies, University of Siena, Siena, Italy; 2 Department of Molecular and Developmental Medicine, University of Siena, Siena, Italy

**Keywords:** Microbiology, Diseases, Health care, Risk factors

## Abstract

The increase in health care-associated infections and antibiotic resistance has led to a growing interest in the search for innovative technologies to solve these problems. In recent years, the interest of the scientific community has focused on violet–blue light at 405 nm (VBL405). This study aimed to assess the VBL405 efficiency in reducing microbial growth on surfaces and air. This descriptive study run between July and October 2020. Petri dishes were contaminated with *P. aeruginosa*, *E. coli*, *S. aureus*, *S. typhimurium*, *K. pneumoniae* and were placed at 2 and 3 m from a LED light source having a wavelength peak at 405 nm and an irradiance respectively of 967 and 497 µW/cm^2^. Simultaneously, the air in the room was sampled for 5 days with two air samplers (SAS) before and after the exposition to the VBL405 source. The highest microbial reduction was reached 2 m directly under the light source: *S. typhimurium* (2.93 log_10_), *K. pneumoniae* (2.30 log_10_), *S. aureus* (3.98 log_10_), *E. coli* (3.83 log_10_), *P. aeruginosa* (3.86 log_10_). At a distance of 3 m from the light source, the greatest reduction was observed for *S. aureus* (3.49 log_10_), and *P. aeruginosa* (3.80 log_10_). An average percent microbial reduction of about 70% was found in the sampled air after 12 h of exposure to VBL405. VBL405 has proven to contrast microbial growth on the plates. Implementing this technology in the environment to provide continuous disinfection and to control microbial presence, even in the presence of people, may be an innovative solution.

## Introduction

Over the last 10 years, scientific evidence suggests that the hospital environment is an important source of transmission of microorganisms that can colonise humans and cause infections^1^. Healthcare-associated infections (HAIs) are a threat to patient's health^2^, especially since they are often caused by poly-chemically resistant bacteria^3^. In a context in which infections caused by bacterial strains (resistant to a broad spectrum of antibiotics) are increasingly widespread and difficult to treat^4^, it is clear that collective infection prevention is an important priority for the scientific community^5^.

Infection control procedures, such as handwashing, air exchange, cleaning of surfaces and medical-surgical devices, are essential to counter the transmission of nosocomial infections^6–8^; however, the growing awareness that the hospital environment can be a source of spreading pathogens has led to a renewed focus on cleaning and disinfection^9^. Consequently, there has been increased interest in the development of new disinfection technologies that, alone or in combination with traditional methods, can ensure the containment of infections^10,11^.

Considerable interest has been directed towards using the antimicrobial properties of light to improve the healthiness of environments^12^.

Ultraviolet radiation (UV) has been used for many years to disinfect air and surfaces. Indeed, the effectiveness of UV-C (wavelength 200-280 nm) disinfection has been widely demonstrated in the hospital environment^13,14^. Recently, the antimicrobial properties of near UV-A, recognised as violet–blue light, have also emerged as a growing research topic. In particular, violet–blue light at 405 nm (VBL405) proved effective for the inactivation of microbial species^15–17^.

The mechanism of action of VBL405 is an oxygen-dependent reaction. Endogenous photo-reactive molecules (mainly porphyrins), naturally present within microorganisms, are photo-excited by light at 405 nm acting as photo-sensitizers. Excitation of porphyrins leads to energy transfer and production of cytotoxic oxygen-derived species, such as singlet oxygen. The reactive oxygen species (ROS) produced interact with cellular structures through the oxidation of macromolecules such as proteins, lipids and nucleic acids. This leads to significant cell damage at different levels in viral, bacterial and fungal cells^16,18,19^.

The great interest for VBL405 is the result of many factors. Several studies have shown that it has a broad spectrum of activity, including antibiotic-resistant bacteria such as MRSA or HCA-associated organisms like *Clostridioides difficile*, *Acinetobacter baumannii, Escherichia coli*, *Staphylococcus epidermidis*, *Pseudomonas aeruginosa*, *Klebsiella pneumoniae*, *Streptococcus pyogenes* and different *Mycobacterium* species^16,20,21^.

Studies on eukaryotic cells have also shown that bacterial cells are more sensitive than mammalian cells, potentially allowing bacterial cells to be inactivated in contaminated tissues while sparing eukaryotic cells^22–24^. Therefore, it could treat infected organic tissue injuries and dynamic disinfection environments^25^. In addition, VBL405 can also be used on delicate sanitary equipment because the natural and synthetic polymers do not undergo significant degradation after prolonged exposure to this wavelength (as happens with UV-C radiation), keeping the durability and mechanical properties of the materials intact^26–28^. These aspects are crucial because they would allow continuous decontamination even in occupied environments when contamination occurs, thus reducing the risk of infection in healthcare and non-healthcare settings^29^.

Reference literature indicates that the disinfection efficacy of VBL405 on pathogens depends both on the intrinsic characteristics of the bacteria, such as species, gram classification and type of endogenous photosensitiser, and on the characteristics of the light source, such as intensity, dose and distance from the source^16,22,30,31^. This latter aspect, and the variability of results associated with the distance of the source from each species studied, was one of the main objectives of our study. Many studies have been carried out in controlled environments, where parameters such as distances, doses and exposure times are different from real ones; this research aims to test whether a lamp equipped with VBL405 technology can reduce microbial contamination in an enclosed environment with a lamp arrangement similar to that achievable in a real setting^31,32^. By determining how the positioning of the source can influence the effectiveness of irradiation in decontamination, we could optimise the calibration of other parameters and establish the applicability of this technology.

## Materials and methods

**Figure 1:**
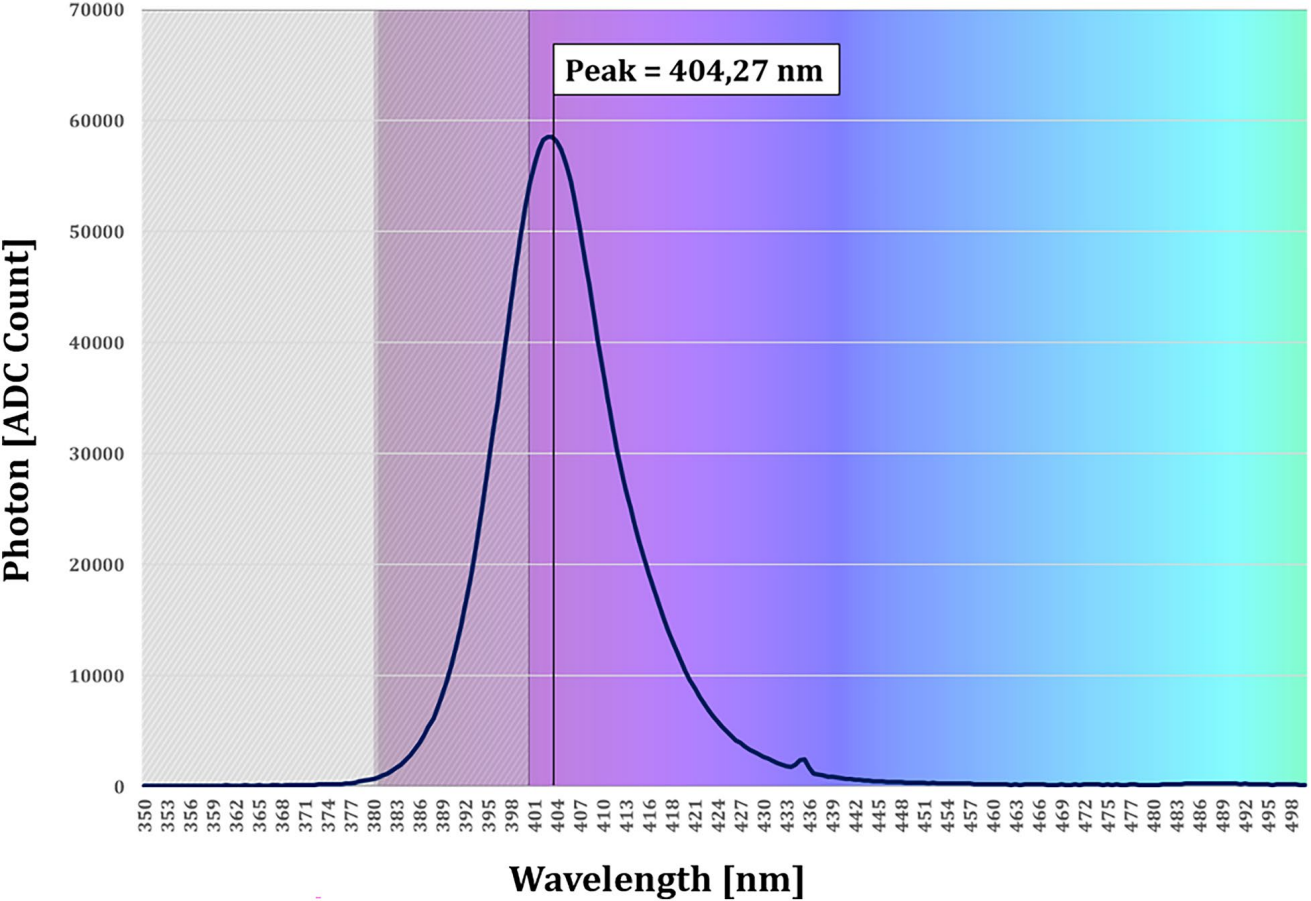
Spectral behaviour of the VBL405 LED: photon counts. Spectral bandwidth 395–415 nm.

This descriptive study was conducted between July and October 2020. Two ceiling lamps, made up of 12 white light-emitting diodes (LEDs) Nichia NVSW219FT (Nichia, Anan, Japan) and 69 VBL405 LEDs, centred at the wavelength of 405 nm (Fig. 1), by Luminus SST-10-UV (Luminus, Sunnyvale, USA) were installed in a room of the Department of Molecular and Developmental Medicine, University of Siena.

**Figure 2:**
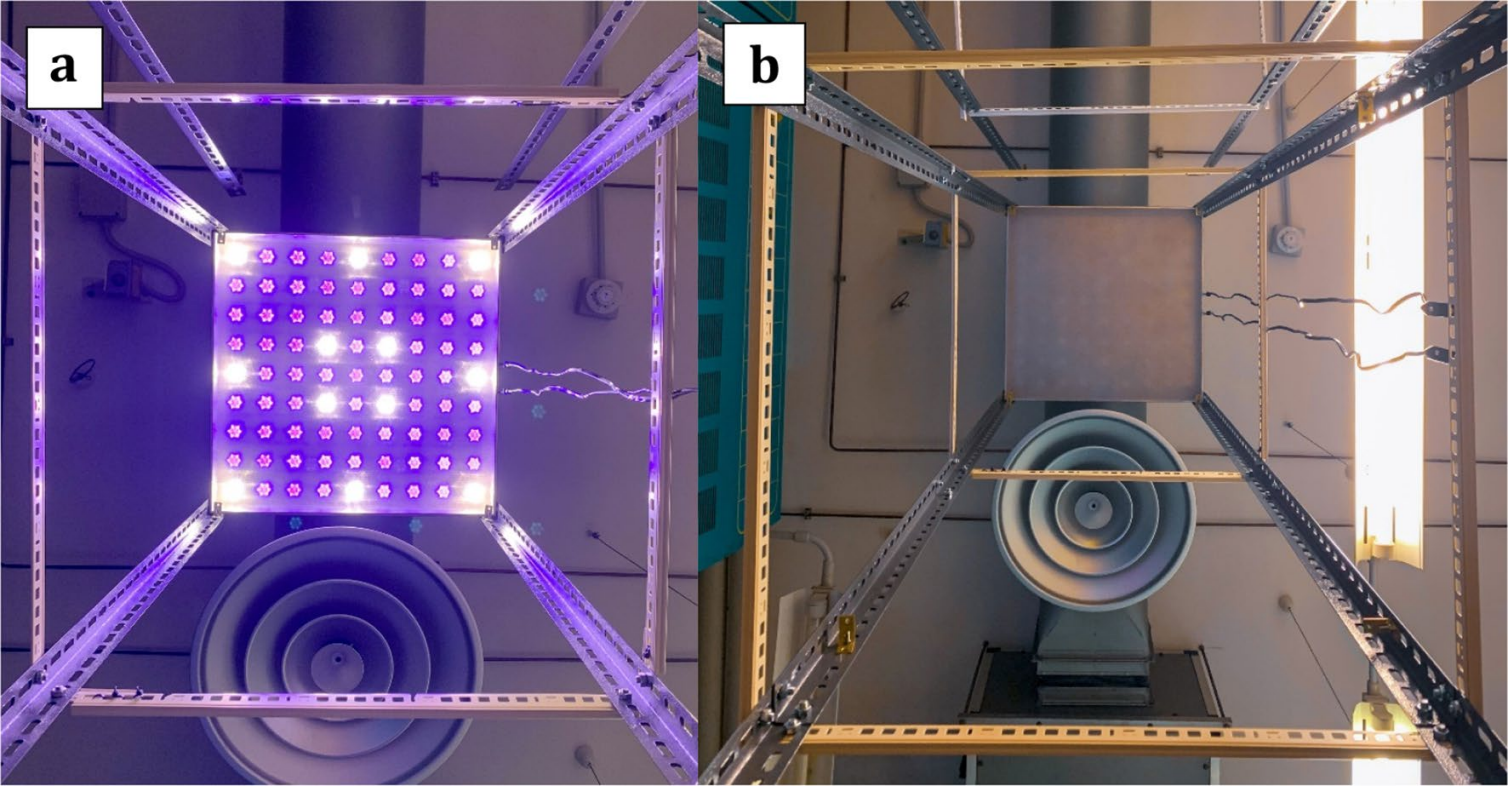
VBL405 ceiling lamp. View from below of the ceiling lamp turned ON (**a**) and OFF (**b**).

The lamps had a Polymethyl methacrylate panel positioned directly under the light source (at a distance of 5 cm from the LEDs) to attenuate and spread the device’s light intensity (Fig. 2a,b).

Photometric characterizations of the lamps were performed using a spectrophotometer Avantes ULS2048CL-EVO (Avantes, Apeldoorn, Netherlands). To evaluate the microbiocidal performance of the VBL405 ceiling lamps, five bacterial strains were selected: *E. coli* (ATCC 8739), *S. aureus* (ATCC 43300), *K. pneumoniae* (ATCC BAA-1705), *P. aeruginosa* (ATCC 27853) and *S. typhimurium* (ATCC 23853).

Each strain was sown on Plate Count Agar (PCA) (Oxoid, Basingstoke, United Kingdom) medium and incubated in a thermostat at 36 °C overnight. Subsequently, some colonies were diluted in Phosphate Buffered Saline (PBS) and measured on the turbidimeter to reach turbidity of 0.5 McFarland, corresponding to approximately 1.5 × 10^8^ Colony Forming Units (CFU) per mL. After scalar dilutions, 100 µL of the inoculum (from an initial concentration of 1.5 × 10^5^ CFU/mL) were transferred into 90 mm Petri dishes (approx. 1.5 × 10^4^ CFU/100µL) with solid culture medium (PCA). The inoculum was spread until dry, inside the laminar flow hood, with a sterile spatula.

**Figure 3:**
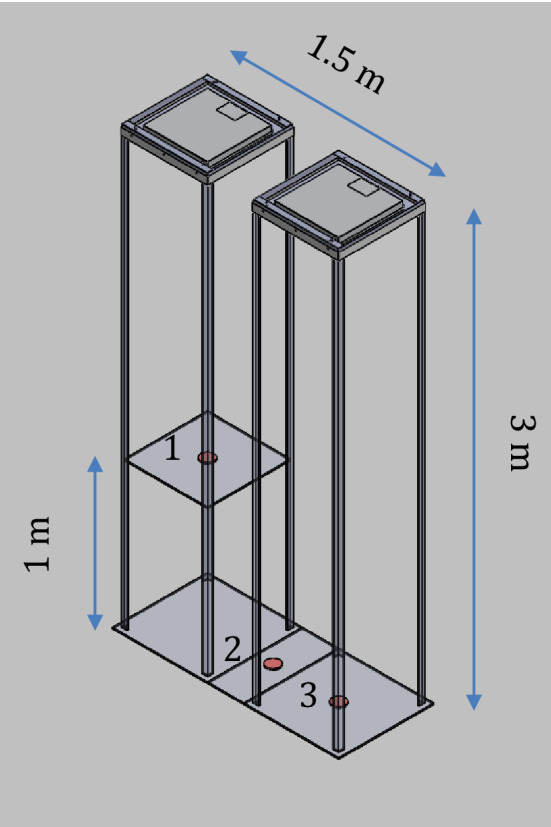
Schematic representation of the experimental setting. The VBL405 LED light sources are located on the top of the structure. Petri dishes are centered in three positions (red circles): (1) 2 m under one lamp; (2) 3 m under the light sources, midway between them; (3) 3 m under the other lamp.

We positioned the contaminated Petri dishes at three different positions from the light source: (i) 2 m directly under one of the light sources, (ii) 3 m between two ceiling lights and (iii) 3 m directly under one of the light sources, having an irradiance respectively of 957 µW/cm^2^, 477 µW/cm^2^ and 497 µW/cm^2^. The absolute dose in J/cm^2^, given by the irradiance (W/cm^2^) and the exposure time (in seconds), was calculated to correlate the bacterial inactivation. All the plates contaminated were exposed for 12 h receiving doses of 41.8 J/cm^2^ in position 1 (2 m directly under one of the light source), 20.6 J/cm^2^ in position 2 (3 m under the light sources, midway between them) and 21.5 J/cm^2^ in position 3 (3 m directly under the other light source) (Fig. 3).

For each sample exposed to the light sources, we prepared a positive control. The positive controls, for the duration of the test, were kept at the same temperature and humidity conditions (approx. 22 °C, with a relative humidity of 23%) as the exposed samples but away from light sources, so that bacterial growth was not impacted. Subsequently, scalar dilutions were prepared and plated on growth medium. At the end of exposure, all samples and their respective positive controls were incubated at 36 °C and counted using a manual colony counter after 48 h. The abatement level of each microorganism were calculated from the logarithmic CFU reduction of the exposed samples with that of the positive controls. The plates were maintained and exposed to light with polystyrene cover on the plate. The attenuation of light through a transparent medium follows an exponential relationship, known as Lambert's law, so the energy doses administered were measured considering the cover attenuation effect of about 7%.

The experiment was repeated in triplicate for each bacterial strain.

In addition, for 5 separate days 2 m^3^ of air from the sealed room were aspirated with Sas Microflow α (Aquaria, Lacchiarella, Italy) samplers, at a speed of 120 L/min. The aspirated air impacted on the surface of 60 mm Ø Petri plates filled with PCA medium. Specifically, the first sampling (T0) was operated one hour after the lamps were turned on and the doors closed, meanwhile the second sampling (T1) was collected immediately after the end of the exposition to the light source (12 h from the experiment start). The plates were then removed and incubated at 36 °C for 48 h to estimate the reduction of the environmental microbial contamination. The first sampling T0 was deliberately started one hour after the lamps were switched on. This was because we wanted to be sure that the microbial composition in the air did not appear elevated just because one or more operators had passed into the room shortly before to prepare the setting for the experiment. In this way, we allowed the levels of microbial contamination in the environment to return to a 'real' level and stabilise, so as not to overestimate any abatement values present at the end of the exposure. During the experiment, the doors and windows of the room were closed, and the ventilation systems were turned off.

### Statistical analysis

All the data collected during the study were entered into a database and included the following variables: Date of test, Petri dish ID, CFUs/mL, CFUs/m^3^, microorganism’s species and inoculum concentrations. Descriptive statistics of empirical data was calculated as mean of base-10 logarithmic (log_10_) CFU reduction together with 95% confidence intervals, to evaluate the reliability of measurements. Statistical comparison of mean log_10_ reductions among the 5 bacteria, by the two VLB405 lamps, was performed using one-way ANOVA, having previously verified with the Kolmogorov–Smirnov test that all sampling data were significantly extracted from a normal distribution. Comparisons between all pairs of bacteria were performed with Bonferroni’s *post-hoc* test, which corrects for statistical significance when performing multiple nonindependent pairwise comparisons. A significance level of 95% was used for all statistical analyses.

The Microsoft Excel software (version 2016) has been used to organize the database. Statistical analysis was performed using Stata software (version 16).

### Simulation model

A photometric simulation was performed using the software Ansys Speos 2022 to model the spatial distribution of VBL405 irradiance on those sections parallel to the floor where the experimental measurement points were located (Fig. 3). Solidworks 2020 CAD software was used to realize the 3D environment. The size of the simulation sectional planes was chosen to be 3.5 × 6.5 m, corresponding to the actual size of the room in which the experiments were carried out.

The following photometric simulation parameters were set:

- radiant flux of VBL405 LEDs = 0.95 W, taken from the LED datasheet as the median value of the intermediate production bin;
- Lambertian type of intensity with an aperture angle of 130°;
- 7% absorption of VBL405 by the polymer cover protecting the LEDs.

## Results

### Experimental results

**Figure 4:**
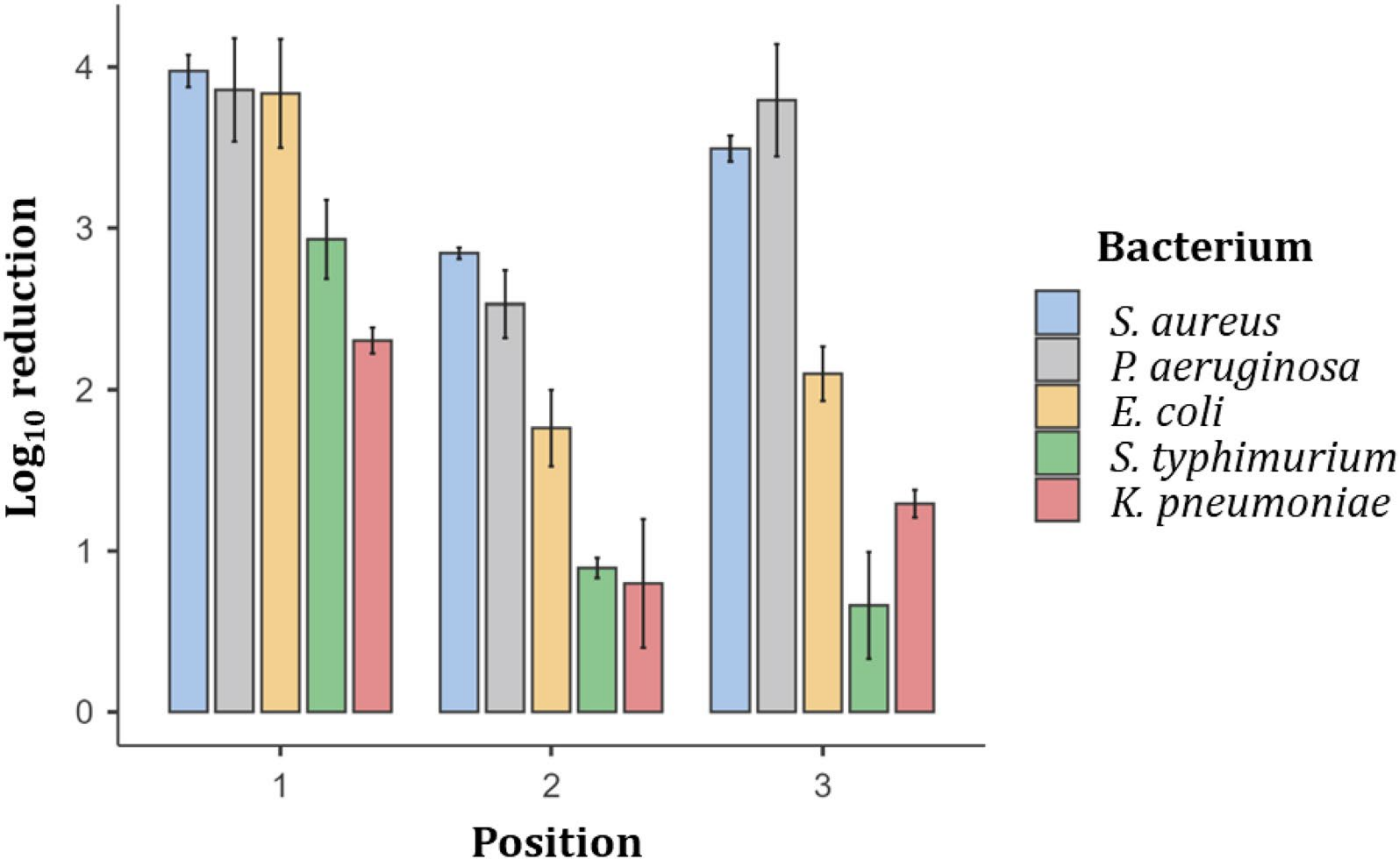
Bar plot of the mean log_10_ reduction of the 5 microbes after 12 h of exposure to VBL405. The vertical intervals across the top of the bars represent ± 1 standard error of mean.

The experiment has shown different results depending on the microorganism and the position of measurement (Table 1 and Fig. 4).

**Table 1:** Microbes log_10_ reduction after 15 h exposure to VBL405.

Position	*S. aureus*		*P. aeruginosa*		*E. coli*		*S. typhimurium*		*K. pneumoniae*	
	Mean	95% CI	Mean	95% CI	Mean	95% CI	Mean	95% CI	Mean	95% CI
1	3.98	3.78–4.17	3.86	3.23–4.49	3.84	3.17–4.50	2.93	2.45–3.41	2.31	2.15–2.46
2	2.84	2.77–2.91	2.53	2.12–2.94	1.76	1.30–2.23	0.89	0.77–1.02	0.80	0.01–1.58
3	3.49	3.33–3.65	3.79	3.11–4.48	2.10	1.77–2.43	0.66	0.01–1.31	1.29	1.13–1.46

The one-way ANOVA statistical analysis and the pairwise comparison between all bacteria performed with Bonferroni's *post-hoc* test (Table 2) show that there are no statistically significant differences between *S. aureus* and *P. aeruginosa* and between *S. typhimurium* and *K. pneumoniae* at all three positions (Table 2). Table 1 and Fig. 4 show higher average log_10_ reduction values in the first two bacteria (around 4 log_10_ at positions 1 and 3 and between 2.5 and 3 log_10_ at position 2), compared to the second pair of bacteria (values between 2 and 3 log_10_ at position 1 and around 1 log_10_ or lower at positions 2 and 3).

**Table 2:** p-values of the one-way ANOVA and of the *post-hoc* Bonferroni test for pairwise comparison of bacteria log_10_ reduction.

ANOVA	Position	Bacteria				Bacteria
		*P. aeruginosa*	*E. coli*	*S. typhimurium*	*K. pneumoniae*	
0.002	1	1.000	1.000	0.123	0.007	*S. aureus*
< 0.001	2	1.000	0.077	0.001	0.001	
< 0.001	3	1.000	0.018	< 0.001	0.001	
	1	–	1.000	0.220	0.011	*P. aeruginosa*
	2	–	0.401	0.005	0.003	
	3	–	0.005	< 0.001	< 0.001	
	1	–	–	0.246	0.012	*E. coli*
	2	–	–	0.235	0.142	
	3	–	–	0.015	0.351	
	1	–	–	–	0.987	*S. typhimurium*
	2	–	–	–	1.000	
	3	–	–	–	0.855	

The differences in the mean log_10_ reductions between each of the bacteria, in one pair and each of those in the other pair, were almost all the time statistically significant (Table 2): only at position 1, at 2 m directly under a lamp (Fig. 3), was the mean log_10_ reduction of *S. typhimurium* not significantly different from that of *S. aureus* and *P. aeruginosa* (ANOVA, p > 0.05).

*Escherichia coli* showed average log_10_ reductions comparable to those of *S. aureus* and *P. aeruginosa* at position 1, and intermediate values at positions 2 and 3, wich were statistically different from *S. aureus*, *P. aeruginosa* and *S. typhimurium* at position 3 and from *K. Pneumoniae* at position 1. No statistically significant differences were found in all comparisons with the other bacteria at position 2 for *E. coli* (ANOVA, p>0.05).

It can also be noted as the two lowest log_10_ reductions are for *K. pneumoniae* in position 2 (0.80) and *S. typhimurium* in position 3 (0.66). Their 95% CIs are very large with lower bounds very close to 0. Although these confidence intervals may decrease as the number of replicates increases, this could also indicate that too low log_10_ reduction mean values tend to be not statistically significant.

The highest microbial reduction rates were observed for all bacterial species at an irradiance value of 957 µW/cm^2^ (position 1). The lowest log_10_ reduction was observed for 4 out of 5 bacterial species at an irradiance of 477 µW/cm^2^ (position 2). For *S. typhimurium*, the lowest bacterial reduction was observed at an irradiance of 497 µW/cm^2^ (position 3).

**Figure 5:**
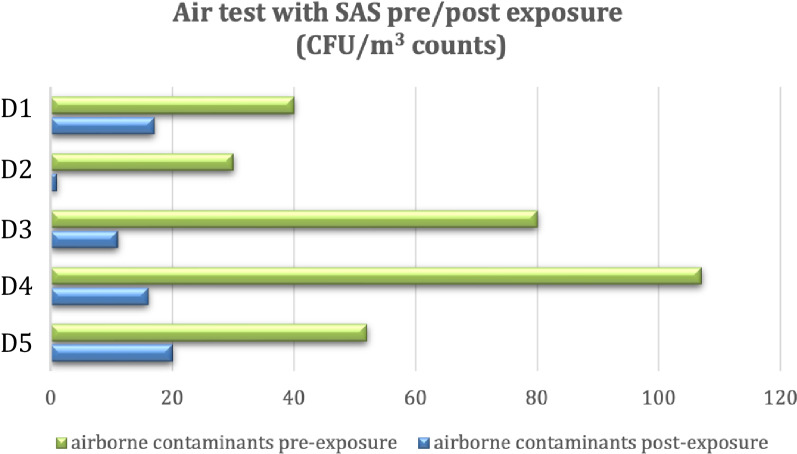
SAS air test. CFU/m^3^ counted after several days (D1-5). Airborne contaminants before (green bars) and after (blue bars) the exposure to VBL405.

From the SAS air tests we observed a constant reduction of airborne microorganisms after 12 h of exposure. We also observed that consecutive exposure to the ceiling lights for more days brought to a general reduction of environmental contamination. The average bacterial count at T0 (1 h after start) over the 5 days of the experiment was 61.8 CFU/m^3^. In contrast, the average value collected at T1 (12 h later) over the 5 days was 13 CFU/m^3^. A decrease of 48.8 CFU/m^3^ was observed, corresponding to a percentage reduction of 79.0% (Fig. 5).

### Simulation results

**Figure 6:**
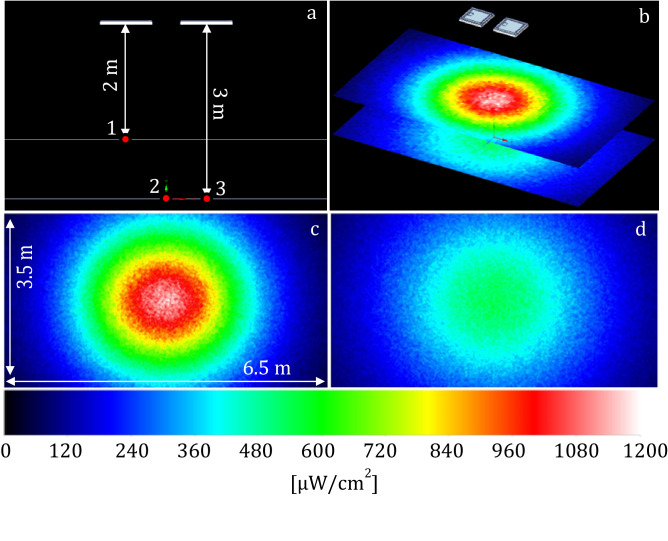
Ansys Speos simulator. (**a**) distance between the measurement and simulation planes and the ceiling where the lamps are located; (**b**) color representation of the irradiance distribution on the simulation planes; (**c**) distribution of irradiance on the first plane (2 m from ceiling); (**d**) distribution of irradiance on the second plane (3 m from ceiling).

Figure 6 shows the simulation results. Based on colors, it is possible to appreciate the distribution of irradiance values, both at the measurement points and over the entire surface of the represented planes.

Table 3 compares the measured and simulated irradiances. The absolute percentage errors are in the narrow range of 2–3%. These errors are fully compatible with fluctuations in the power of the different LEDs and variations in the optical properties (absorption, reflection, scattering, etc.) of the materials in the experimental setting.

**Table 3:** Fitting of simulated to measured irradiances.

Position	Measured irradiance (µW/cm^2^)	Simulated irradiance (µW/cm^2^)	Absolute error (%)
1	957	979	2.25
2	477	488	2.37
3	497	481	3.28

## Discussion

The importance of the hospital environment as a source of nosocomial pathogens has focused on conventional cleaning and disinfection procedures and the development of new technologies, including UV systems, steam cleaning hydrogen peroxide vapour and super-oxidized water fogging^33^. While demonstrating very good results, these last systems require, for safety reasons, the supervision of an experienced operator and, at the time of use, unoccupied rooms. This leads to a higher waiting time in crowded wards and a significant economic expense. Although these systems result in effective disinfection, another study has reported rapid recontamination of the room once the treatment is over^29^. Nevertheless, these methods are suitable for terminal and deep cleanings.

This study showed that 405 nm light has a relevant bactericidal effect on several important and taxonomically diverse bacterial pathogens.

The bacteria most resistant to VLB405 radiations were *S. typhimurium* and *K. pneumoniae*: ANOVA analysis showed systematic significant lower log_10_ reductions singularly compared with both *S. aureus* and *P. aeruginosa* and, at a distance of 3 m (positions 2 and 3), in some comparisons also with *E. coli* (see Tables 1, 2 and Fig. 4).

Different abatement rate among bacterial strains may be explained by the different biological dispositions and expressions of endogenous photosensitizers in different species^20^.

As explained by Kim et al., cellular sensitivity to VBL405 also varies with bacterial strain and serotype. The extent of cellular damage could explain the different susceptibility of different bacterial species. Indeed, it would appear that the small amounts of endogenous porphyrins in *S. typhimurium* cells do not generate sufficient ROS to disrupt the integrity of the cell membrane during LED illumination^34^. However, these might be sufficient to oxidize DNA and affect efflux pump activity and glucose uptake systems^35^.

In addition, different bacterial species produce different porphyrins, and because peak absorption wavelengths might vary between different photosensitizers, different wavelengths may be required for optimal photo-stimulation^16,36^.

Comparing the results obtained with data in the literature, it can be seen that for similar doses the results are variable. This can be explained to the many factors that condition the context of this type of study. *Maclean *et al. obtained bacterial colony abatements of 3.9 log_10_, 3.1 log_10_ and 4.2 log_10_ for *K. pneumoniae*, *E. coli* and *P. aeruginosa*, respectively, at doses of 180 J/cm^2^. A 5-log_10_ abatement was achieved at only 36 J/cm^2^ for *S. aureus*^16^. At doses of 41.8 J/cm^2^ we found logarithmic reductions of 2.3 log_10_ for *K. pneumoniae*, 3.8 log_10_ for *E. coli*, 3.9 log_10_ for *P. aeruginosa* and 4 log_10_ for *S. aureus*. *Shehatou *et al. obtained for all four bacteria a 4-log_10_ abatement with irradian values ranging from 72 to 216 J/cm^2^^30,37^. Therefore, the results obtained for *S. aureus*, *K. pneumoniae* and *E. coli* are similar with those found in the literature, with similar doses for killing the same number of CFU or higher doses for a larger number of colonies. In contrast, for *P. aeruginosa*, our results are discordant with most in the literature, although there are several studies that disagree with each other^16,30,37^. The same is for *S. typhimurium,* where much higher dosages are found necessary to kill the same number of colonies. This can be explained if different strains may respond differently to the same dosage of VBL405 and that environmental conditions and culture media may influence the response to irradiation^38^.

This is consistent with observations made by Boyce et al*.* showing that the antimicrobial effect of ultraviolet radiation is dependent on distance and source orientation, with significant differences and reduction in efficacy as distance increases^31^.

Simulation data approximated well experimental data, in all the three measurement points. Errors between simulated and measured irradiances were very low (around 2–3%), demonstrating the simulation reliability in representing real data. Therefore, the Ansys Speos simulator can be used to design accurately any 3D VBL405 lighting scenario, with any number and arrangement of lamps*.* The microbicidal effects combined with the associated light energy, allow the simulator to estimate the reachable disinfection levels for each bacterium in the different areas of the illuminated volume. In addition, simulations allow the optimisation of spatial light distribution. From a legislative perspective, this is an important aid to control the safety limits for operators, which were defined by national and international scientific and technical committees.

Indeed, violet–blue wavelengths within the visible spectrum can cause harmful effects at high irradiance levels especially at 440 nm, which can cause photoretinitis, and 480 nm, which is the peak sensitivity of mammalian photosensitive retinal ganglion cells, inducing diverse physiological responses to light including circadian physiology and pupil constriction^39^. Although 405 nm light is germicidal, it falls within a relatively benign wavelength region, and, if operated at appropriately low irradiance levels, it is safe for human exposure^40^. Therefore, this disinfection technology can be used continuously in the presence of people, thus facilitating a background disinfection to maintains low levels of contaminations even in healthcare^26^. The use of 405 nm light technology can improve the disinfection levels achieved with periodic cleaning, as it can also be used on delicate sanitary equipment^27^. This is not possible with UV radiation because natural and synthetic polymers undergo significant degradation after prolonged exposure. In general, UV rays adversely affect the mechanical properties of materials such as plastics and wood, limiting their lifespan^28^.

A comparative study of the degradative effects of UV radiation and 405 nm sources on flexible endoscopes showed that germicidal ultraviolet radiation leads to device failure and an increased risk of infection for patients, unlike 405 nm light. Polymers absorb UV radiation, resulting in photo-degradation, bond splitting, and chemical transformations that create structural heterogeneity. The loss of product characteristics and properties reduces the durability of the endoscope and leads to an increased risk of infection for the patient. Cracks, which form as a result of photo-degradation, can increase biofouling and inhibit proper cleaning of the instrument. Exposure to UV-C light showed increased adhesion of *P. aeruginosa*, an effect which is not observed with 405 nm^41^.

In addition, continuous cleaning of rooms in the presence of healthcare workers and patients is not possible with UV radiation as chronic exposure to these wavelengths may cause photo-ageing, immunosuppression and carcinogenesis in mammalian cells^42^. Mammalian cells and bacteria have a different sensitivity to 405 nm light. Studies on osteoblasts suggest that exposure of cells to 405 nm light up to a dose of 36 J∕cm^2^ causes no observable effects on cell viability, function, proliferation rate and morphology.

When studying the effects of exposure to 405 nm light on bacterial cells, results prove that the same doses induce significant bactericidal effects^15,22^.

It needs to be reiterated that 405 nm light technology should be used to integrate classical cleaning and disinfection methods, thus providing an additive effect, and not to replace traditional techniques.

A limitation of all disinfection systems that use light is that only surfaces directly or reflectively exposed to light can be treated, and the effects on occluded or shaded areas are limited, although VBL405 is better able than UV light to pass through transparent surfaces.

Decontamination of spores (e.g. of *C. difficile*) requires a much higher VBL405 irradiance in the relatively short time that people are present^43^. However, a significant improvement in inactivation could be achieved by combining VBL405 with other decontamination methods such as photocatalytic and oxidative biocides^17^.

Another limitation of LED technology with 405 nm emission is that the light peaks generated at this frequency are not equal. When the peak is at 405 nm, left-handed tails are generated that can drop into the ultraviolet spectrum, generating photo-biologically hazardous energy. According to EN 62471 standards, it is important to assess the magnitude of this energy to ensure safe technology. LED irradiance can be monitored and controlled through appropriate UV sensors to reduce it below safe limits, but LEDs with somewhat longer peak wavelengths, such as 415 nm, can also be used, having similar disinfection ability. In this way, the tail does not fall on the UV spectrum or at least the UV percentage in the light beam is so low that it is not associated with photo-biological risks. In any case, it may be useful to attenuate the power of the sources in the presence of people, but operate them at full power in the absence of individuals by significantly reducing their action times^44^.

Regarding the experimental laboratory aspects, our study presents some limitations.

Plate Count Agar is a growth medium for bacteria, which will therefore be metabolically active. However, given the temperature, humidity and room conditions, these should not have had a significant effect on the metabolic response of the bacteria. It can therefore be assumed that the conditions under which the bacteria were kept did not lead to an overestimation or an underestimation of the effect of VBL405.

The experiments were conducted in a closed environment. In a real situation, multiple variables could influence the test results. First of all, the investigation was carried out under conditions of no airflow, with windows and doors closed. In a real context, air circulation would be sufficient to reduce the bacterial count in the room so that the results could be different. To our knowledge, the effectiveness of VBL405 irradiation as a disinfection method has never been tested in a room with constant air recirculation. Furthermore, most of the experiments have been conducted in vitro; bacteria in a real environment are numerous and characterised by high biodiversity. Therefore, irradiation responses may differ between species due to both different genetic codes and potential adaptation mechanisms developed under particular habitat conditions. Consequently, it would be helpful to evaluate the efficacy of lamps with 405 nm LED sources in a real environment.

Experiments with VBL405 ceiling lamps on contaminated plates showed that microorganisms have different resistance against this type of radiation. Based on preliminary investigations, VBL405 has proven to contrast microbial contamination growth on the plates, although with different effects at different distances from the light source. The bacteria used in this study were produced under laboratory cultivation conditions; therefore, they may differ from bacteria found in a natural environment, especially physiological status, biological set-up and stress conditions. It would also be interesting to determine if the bacteria exposed to natural lighting have developed protective mechanisms to resist the inactivating effects of these wavelengths.

In this systems, it is very important to take into consideration many parameter such as LED power, source distance and all the interfering elements. The proper configuration of the entire system is crucial for optimal irradiance effectiveness: shaded areas, screens, and panels can significantly reduce the microbiocidal performance of the ceiling lamp. However, in general, the distance from the source remains one of the main determinants of the effectiveness of disinfection with VBL405. In particular, the most significant reduction (4 log_10_ reduction) in the bacterial load was reached for four strains located in the nearest position to the lamp (2 m under ceiling lamps).

Air tests, carried out for 5 days, showed a constant reduction of airborne contaminants in the exposure room. In particular, using the ceiling lamp on consecutive days has relevant effects on air quality.

In conclusion, the ceiling lamp can be efficiently used to reduce microbial growth when the following parameters that influence the environment are properly managed: (i) the exposure time, (ii) the light power, (iii) the number and positioning of light sources, (iv) the distance from the light sources, (v) the obstacles along the light paths.

## Data Availability

The datasets used and/or analysed during the current study available from the corresponding author on reasonable request.

## Abbreviations

VBL405: Violet–blue light at 405 nm
nUV-A: Near UV-A
LED: Light emitting diode
SAS: Surface air system
HAI: Healthcare-associated infections
MRSA: Methicillin-resistant *Staphylococcus* *aureus*
VRE: Vancomycin-resistant *Enterococcus*
MDR: Multidrug-resistant
UV: Ultraviolet
HCA: Community-onset healthcare-associated
PCA: Plate count agar
PBS: Phosphate buffered saline
CFU: Colony forming unit
ROS: Reactive oxygen species

## References

[1] Weiner-Lastinger LM, Abner S, Edwards JR, Kallen AJ, Karlsson M, Magill SS (2020). Antimicrobial-resistant pathogens associated with adult healthcare-associated infections: Summary of data reported to the National Healthcare Safety Network, 2015–2017. Infect. Control Hosp. Epidemiol..

[2] Cassini A, Plachouras D, Eckmanns T, Abu Sin M, Blank HP, Ducomble T (2016). Burden of six healthcare-associated infections on European population health: Estimating incidence-based disability-adjusted life years through a population prevalence-based modelling study. PLoS Med..

[3] Friedman ND, Temkin E, Carmeli Y (2016). The negative impact of antibiotic resistance. Clin. Microbiol. Infect..

[4] Sabtu N, Enoch DA, Brown NM (2015). Antibiotic resistance: What, why, where, when and how?. Br. Med. Bull..

[5] Collins AS. Preventing health care–associated infections. In *Patient Safety and Quality: An Evidence-Based Handbook for Nurses* (ed Hughes, R. G.) (Agency for Healthcare Research and Quality (US), 2008) (Advances in Patient Safety) http://www.ncbi.nlm.nih.gov/books/NBK2683/. 21328752

[6] Messina G, Quercioli C, Burgassi S, Nisticò F, Lupoli A, Nante N (2011). How many bacteria live on the keyboard of your computer?. Am. J. Infect. Control..

[7] Rutala WA, Weber DJ (2016). Monitoring and improving the effectiveness of surface cleaning and disinfection. Am. J. Infect. Control..

[8] Carling PC, Bartley JM (2010). Evaluating hygienic cleaning in health care settings: What you do not know can harm your patients. Am. J. Infect. Control..

[9] Stilo A (2016). Hand washing in operating room: a procedural comparison. Epidemiol. Biostat. Public Health..

[10] Weber DJ, Rutala WA, Anderson DJ, Chen LF, Sickbert-Bennett EE, Boyce JM (2016). Effectiveness of ultraviolet devices and hydrogen peroxide systems for terminal room decontamination: Focus on clinical trials. Am. J. Infect. Control..

[11] Deshpande A, Mana TSC, Cadnum JL, Jencson AC, Sitzlar B, Fertelli D (2014). Evaluation of a sporicidal peracetic acid/hydrogen peroxide-based daily disinfectant cleaner. Infect. Control Hosp. Epidemiol..

[12] Messina G, Amodeo D, Corazza A, Nante N, Cevenini G (2022). Analysis of the physical and microbiocidal characteristics of an emerging and innovative UV disinfection technology. BMJ Innov..

[13] Messina G, Burgassi S, Messina D, Montagnani V, Cevenini G (2015). A new UV-LED device for automatic disinfection of stethoscope membranes. Am. J. Infect. Control..

[14] Ramos CCR, Roque JLA, Sarmiento DB, Suarez LEG, Sunio JTP, Tabungar KIB (2020). Use of ultraviolet-C in environmental sterilization in hospitals: A systematic review on efficacy and safety. Int. J. Health Sci..

[15] Ramakrishnan P, Maclean M, MacGregor SJ, Anderson JG, Grant MH (2014). Differential sensitivity of osteoblasts and bacterial pathogens to 405-nm light highlighting potential for decontamination applications in orthopedic surgery. J. Biomed. Opt..

[16] Maclean M, MacGregor SJ, Anderson JG, Woolsey G (2009). Inactivation of bacterial pathogens following exposure to light from a 405-nanometer light-emitting diode array. Appl. Environ. Microbiol..

[17] Maclean M, Murdoch LE, MacGregor SJ, Anderson JG (2013). Sporicidal effects of high-intensity 405 nm visible light on endospore-forming bacteria. Photochem. Photobiol..

[18] McKenzie K, Maclean M, Grant MH, Ramakrishnan P, MacGregor SJ, Anderson JG (2016). The effects of 405 nm light on bacterial membrane integrity determined by salt and bile tolerance assays, leakage of UV-absorbing material and SYTOX green labelling. Microbiology.

[19] Maclean M, MacGregor SJ, Anderson JG, Woolsey GA (2008). The role of oxygen in the visible-light inactivation of *Staphylococcus aureus*. J. Photochem. Photobiol. B..

[20] Feyissa Q, Xu F, Ibrahim Z, Li Y, Xu KL, Guo Z (2021). Synergistic bactericidal effects of pairs of photosensitizer molecules activated by ultraviolet A light against bacteria in plasma. Transfusion.

[21] Murdoch LE, Maclean M, Endarko E, MacGregor SJ, Anderson JG (2012). Bactericidal effects of 405 nm light exposure demonstrated by inactivation of *Escherichia, Salmonella, Shigella, Listeria, and Mycobacterium* species in liquid suspensions and on exposed surfaces. Sci. World J..

[22] Dai T, Gupta A, Huang YY, Yin R, Murray CK, Vrahas MS (2013). Blue light rescues mice from potentially fatal *Pseudomonas aeruginosa* burn infection: Efficacy, safety, and mechanism of action. Antimicrob. Agents Chemother..

[23] Bauer R, Hoenes K, Meurle T, Hessling M, Spellerberg B (2021). The effects of violet and blue light irradiation on ESKAPE pathogens and human cells in presence of cell culture media. Sci. Rep..

[24] Stewart CF, Tomb RM, Ralston HJ, Armstrong J, Anderson JG, MacGregor SJ (2022). Violet-blue 405-nm light-based photoinactivation for pathogen reduction of human plasma provides broad antibacterial efficacy without visible degradation of plasma proteins. Photochem. Photobiol..

[25] Maclean M, McKenzie K, Anderson JG, Gettinby G, MacGregor SJ (2014). 405 nm light technology for the inactivation of pathogens and its potential role for environmental disinfection and infection control. J. Hosp. Infect..

[26] Bache SE, Maclean M, MacGregor SJ, Anderson JG, Gettinby G, Coia JE (2012). Clinical studies of the High-Intensity Narrow-Spectrum light Environmental Decontamination System (HINS-light EDS), for continuous disinfection in the burn unit inpatient and outpatient settings. Burns.

[27] Maclean M, Booth M, Anderson J, MacGregor S, Woolsey G, Coia J (2013). Continuous decontamination of an intensive care isolation room during patient occupancy using 405 nm light technology. J. Infect. Prev..

[28] Andrady AL, Hamid SH, Hu X, Torikai A (1998). Effects of increased solar ultraviolet radiation on materials. J. Photochem. Photobiol. B..

[29] Hardy KJ, Gossain S, Henderson N, Drugan C, Oppenheim BA, Gao F (2007). Rapid recontamination with MRSA of the environment of an intensive care unit after decontamination with hydrogen peroxide vapour. J. Hosp. Infect..

[30] Shehatou C, Logunov SL, Dunman PM, Haidaris CG, Klubben WS (2019). Characterizing the antimicrobial properties of 405 nm light and the corning^®^ light-diffusing fiber delivery system. Lasers Surg. Med..

[31] Boyce JM, Farrel PA, Towle D, Fekieta R, Aniskiewicz M (2016). Impact of room location on UV-C irradiance and UV-C dosage and antimicrobial effect delivered by a mobile UV-C light device. Infect. Control Hosp. Epidemiol..

[32] Nerandzic MM, Thota P, Sankar CT, Jencson A, Cadnum JL, Ray AJ (2015). Evaluation of a pulsed xenon ultraviolet disinfection system for reduction of healthcare-associated pathogens in hospital rooms. Infect. Control Hosp. Epidemiol..

[33] French GL, Otter JA, Shannon KP, Adams NMT, Watling D, Parks MJ (2004). Tackling contamination of the hospital environment by methicillin-resistant *Staphylococcus aureus* (MRSA): A comparison between conventional terminal cleaning and hydrogen peroxide vapour decontamination. J. Hosp. Infect..

[34] Kim MJ, Yuk HG (2017). Antibacterial mechanism of 405-nanometer light-emitting diode against salmonella at refrigeration temperature. Appl. Environ. Microbiol..

[35] Kumar A, Ghate V, Kim MJ, Zhou W, Khoo GH, Yuk HG (2015). Kinetics of bacterial inactivation by 405nm and 520nm light emitting diodes and the role of endogenous coproporphyrin on bacterial susceptibility. J. Photochem. Photobiol. B..

[36] Nitzan Y, Salmon-Divon M, Shporen E, Malik Z (2004). ALA induced photodynamic effects on Gram positive and negative bacteria. Photochem. Photobiol. Sci..

[37] Halstead FD, Thwaite JE, Burt R, Laws TR, Raguse M, Moeller R (2016). Antibacterial activity of blue light against nosocomial wound pathogens growing planktonically and as mature biofilms. Appl. Environ. Microbiol..

[38] Kim MJ, Mikš-Krajnik M, Kumar A, Yuk HG (2016). Inactivation by 405 ± 5 nm light emitting diode on *Escherichia coli* O157:H7, *Salmonella Typhimurium*, and *Shigella **sonnei* under refrigerated condition might be due to the loss of membrane integrity. Food Control.

[39] Foster RG (2009). The ‘Third’ photoreceptor system of the eye—photosensitive retinal ganglion cells. Eur. Ophthalmic Rev..

[40] International Commision on Non-Ionizing Radiation Protection (ICNIRP). Guidelines on limits of exposure to ultraviolet radiation of wavelenghts between 180 nm and 400 nm (incoherent radiation). *Health Phys. ***87**(2), 171–186 (2004). 10.1097/00004032-200408000-00006 15257218

[41] Irving D, Lamprou DA, Maclean M, MacGregor SJ, Anderson JG, Grant MH (2016). A comparison study of the degradative effects and safety implications of UVC and 405 nm germicidal light sources for endoscope storage. Polym. Degrad. Stab..

[42] Matsumura Y, Ananthaswamy HN (2004). Toxic effects of ultraviolet radiation on the skin. Toxicol. Appl. Pharmacol..

[43] Djouiai B, Thwaite JE, Laws TR, Commichau FM, Setlow B, Setlow P (2018). Role of DNA repair and protective components in *Bacillus subtilis* spore resistance to inactivation by 400-nm-wavelength blue light. Appl. Environ. Microbiol..

[44] European Standard EN 62471, Photobiological safety of lamps and lamp systems. IEC 62471 (2008).

